# Genitourinary and Syphilitic Diseases

**Published:** 1903-07

**Authors:** Byron H. Daggett

**Affiliations:** Buffalo, N. Y.


### PROGRESS IN MEDICAL SCIENCE.

### Genitourinary and Syphilitic Diseases.

Conducted by BYRON H. DAGGETT, M. D., Buffalo, N. Y.

### BLADDER DRAINAGE.

Reginald Harrison (*Polyclinic*, London, September 17, 1902). Abstract. The power to completely empty and drain the bladder voluntarily of its contents is a natural act, which cannot be regularly averted without exposure to some risk.

1. What disorders may result from imperfect drainage by natural efforts?
2. How may artificial drainage be best effected?
3. What conditions call for the regular use of the catheter?

In children nature has provided for bladder drainage as perfectly as possible. In adults of middle life there are seldom any alterations of structure which interfere with drainage, except stricture or abnormal growths. In more advanced life, when the sixties are reached or before, there is a tendency to some sinking or relaxation of the bladder wall, which renders drainage or emptying the bladder imperfect. This condition, causing incomplete drainage of the bladder, is not uncommon about the age of 60, though physical evidence of prostatic enlargement may be still wanting.

Apart from the changes occurring at different periods of life there are disorders directly due to undrained or stagnant urine. Apart from disturbances such as frequent urination and overflow of urine, there are a number of abnormal urinary conditions which may be classified as (1) intravesical, or those happening within the urinary apparatus; and (2) extravesical, or those due to disturbance of structures in relation to, but outside of the urinary tract.

The intravesical disorders are cystitis, suppuration and ammoniacal decomposition, causing dilatation of the ureters and pelves of the kidneys, distortion of the bladder by formation of trabeculæ, pouches and sacs. By retention and decomposition of the urine the urinary mucosa may become suppurating surfaces. Phosphatic stone may form in the undrained bladder; it is very prone to reform in residual urine.

Extravesical effects of undrained urine: extravasation of urine into the tissues—acute and chronic. (To be considered later.)

Bladder drainage is usually practised by means of catheters. Of these there are a large variety, and they should not be used without professional advice and caution necessary in their employment. Catheters should not be used in place of bougies for dilating strictures or for preventing their recurrence, lest the expulsion powers of the bladder be impaired. Sometimes catheter drainage and irrigation are inefficient, and the bladder must be opened either through the perineum or above the pubis, if the obstruction be due to an intravesical growth; otherwise the perineal route is the one of choice.

As a temporary measure for the relief of overdistension and in other conditions, catheterisation is proper, but in cases of a moderate degree of residual urine from senile prostate or other causes, one should be guarded lest catheterisation, once began, ends only with death. The presence of residual urine does not call for catheterisation, unless the patient be suffering from straining or frequent acts of masturbation. Rising once or twice at night, and corresponding calls during the day, do not warrant interference by catheterisation, lest catheter life be established. Incontinence and decomposition make a necessity of drainage by catheter or otherwise.

